# Novel insights into palatal shelf elevation dynamics in normal mouse embryos

**DOI:** 10.3389/fcell.2025.1532448

**Published:** 2025-02-11

**Authors:** Jeremy P. Goering, Michael Moedritzer, Marta Stetsiv, Dona Greta Isai, Brittany M. Hufft-Martinez, An J. Tran, Zaid Umar, Madison K. Rickabaugh, Paul Keselman, Munish Chauhan, Pamela V. Tran, William M. Brooks, Kenneth J. Fischer, Andras Czirok, Irfan Saadi

**Affiliations:** ^1^ Department of Cell Biology and Physiology, University of Kansas Medical Center, Kansas City, KS, United States; ^2^ Hoglund Biomedical Imaging Center, University of Kansas Medical Center, Kansas City, KS, United States; ^3^ Department of Neurology, University of Kansas Medical Center, Kansas City, KS, United States; ^4^ Department of Mechanical Engineering, University of Kansas, Lawrence, KS, United States; ^5^ Department of Biological Physics, Eotvos University, Budapest, Hungary; ^6^ Institute for Reproductive and Developmental Sciences, University of Kansas Medical Center, Kansas City, KS, United States

**Keywords:** embryonic development, palatogenesis, palatal shelf elevation, cleft palate, embryonic sex difference

## Abstract

Development of the embryonic palate requires that the palatal shelves (PS), which extend from maxillary processes, to grow bilaterally and vertically alongside the tongue. This growth continues until embryonic day (E) 13.5, after which the PS elevate above the tongue and adhere, completing the process by E14.5. Current models indicate that this elevation process involves a complex vertical-to-horizontal PS reorientation. While earlier studies have implied that reorientation occurs rapidly, the precise timing has not been resolved. Time-restricted pregnancies with a 1-h resolution showed that in 97% of C57BL/6J embryos, the PS were unelevated at E14.0. However, 6 h later, at E14.25, the PS had completed elevation in 80% of embryos, indicating that the PS elevate in a rapid and constrained timeframe. Interestingly, all E14.25 embryos with unelevated PS (20%) were female, suggesting sex differences in C57BL/6J PS elevation. In FVB/NJ embryos, the elevation window started earlier (E13.875-E14.25), and without any sex differences. An intermediate stage with unilateral PS elevation was frequently observed. Magnetic resonance imaging (MRI) of various stages showed that PS elevation began with posterior bilateral bulges, which then progressed laterally and anteriorly over time. During elevation, we observed increased cell proliferation in the PS lingual region. Within the bulge, cell orientation was tilted towards the tongue, and actomyosin activity was increased, which together may participate in horizontal projection of the bulge. Thus, our data reveal novel insights into rapid dynamic changes during PS elevation, and lay the foundation for future studies of normal and abnormal palatogenesis.

## Introduction

Clefts of the lip and palate constitute the majority of craniofacial malformations, and collectively affect ∼1 in 800 births worldwide (Mossey et al., 2009; Mossey and Modell, 2012). Clefts of the palate alone affect ∼1 in 1700 births, and are more common in females than males (Mossey et al., 2003; Mossey et al., 2009). Approximately half of cleft palate cases are isolated or nonsyndromic occurrences that have a complex etiology, resulting from both genetic and environmental factors (Jugessur et al., 2009; Dixon et al., 2011; Mangold et al., 2011; Leslie et al., 2015; Leslie et al., 2016; Martinelli et al., 2020). The contribution of these factors has been extensively studied using rodent models over the past 6 decades. The initial focus of these studies was on environmental factors and chemical compounds, as well as on susceptible murine backgrounds and spontaneous mouse mutants (Trasler, 1960; Greene and Kochhar, 1973; 1975; Juriloff, 1980; Slavkin and Melnick, 1982; Pratt et al., 1984a; Pratt et al., 1984b; Vekemans and Biddle, 1984).

Another early focus was to characterize normal palatogenesis. Classical studies identified three main steps in palatogenesis following induction of palatal shelves (PS): 1) vertical PS growth next to the tongue, 2) PS elevation above the tongue, and 3) PS fusion in the midline (Walker and Fraser, 1956; Johnston et al., 1975; Ferguson, 1988; Diewert and Wang, 1992; Gritli-Linde, 2007). These steps were confirmed in humans through fetal analysis and improved ultrasound imaging, making them a fundamental part of most embryology textbooks (Burdi, 1965; Burdi and Silvey, 1969; Diewert, 1983; 1985; Diewert and Shiota, 1990).

Among these three steps, the second, PS elevation, has remained enigmatic. Early studies of PS elevation suggested that the PS performed a simple rotation from a vertical to a horizontal position, or that elevation occurred by vertical resorption and horizontal growth with cell proliferation. These models were challenged by Walker and Fraser (Walker and Fraser, 1956), who showed that physical changes during PS elevation were incompatible with a simple rotation, and that the process was too rapid–less than 2 min with manual manipulation–for cell proliferation to be the main driver of elevation. They instead argued that the PS moved from a vertical to a horizontal position via a process of reorientation. They did acknowledge that cell growth was necessary to build up the force necessary for PS elevation. They further proposed that PS elevation occurred in a developmental window approximately 3 h long, but noted that this window was shifted in different mouse strains.

Since these early studies, there have been few attempts to study normal PS elevation, though some further insight has been gained from the few mouse mutants that appeared to affect PS elevation. The current understanding of PS elevation was comprehensively summarized by Bush and Jiang (Bush and Jiang, 2012). According to the model they compiled, the anterior-most regions of the PS elevate by rotating, while the middle and posterior PS regions elevate by reorienting from a vertical to a horizontal position, as also proposed by Walker and Fraser (1956). These determinations were based on the location of the medial edge epithelium (MEE), which is the region of the PS epithelium that eventually meets and fuses at the midline following elevation. Prior to elevation, the MEE in the anterior-most region of the PS lies at the ventral tip of each vertical shelf. In contrast, the MEE in the middle and posterior regions of the PS lies on the lateral surface. Several pieces of evidence suggest that the vertical-to-horizontal reorientation initiates with the formation of a bulge that extends medially above the tongue. Bush and Jiang (2012) also noted Walker and Fraser’s assertion that this remodeling may proceed unilaterally–that is, with one PS reorienting at a time—as unilateral PS elevation was occasionally observed in normal mouse embryos.

We recently reported that loss of *Specc1l*, which encodes a cytoskeletal actin-regulating protein (Saadi et al., 2023), delayed PS elevation but did not prevent the PS from eventually elevating and fusing (Hall et al., 2020; Goering et al., 2021b). A delay in PS elevation provides a model for isolated cleft palate where the delay can be considered a sensitized background that combined with additional negative genetic or environmental factors, may put the affected individual above the threshold for isolated cleft palate.

While studying the delayed PS elevation in *Specc1l* mutant embryos using overnight matings, we noticed significant inter-litter variability in the staging of the PS, and felt a need to better understand normal PS elevation. Thus, in the present study we performed a careful analysis of two commonly used murine strains (C57BL/6J and FVB/NJ), using time-restricted mating to assess embryonic development at 3-h intervals. We observed that: 1) the PS could elevate in less than 3 h, 2) the PS elevation window is influenced by mouse strain and sex differences, and 3) vertical-to-horizontal remodeling occurs with dynamic lateral anteroposterior changes originating in the posterior PS. We also generated data on cell proliferation, cell orientation, and myosin activity in the PS during elevation. These results build upon the existing model of PS elevation and provide critical new insights into the timing of tissue- and cell-level changes that will not only help understand normal PS elevation, but also help characterize PS elevation defects in existing and novel mutant mouse models.

## Materials and methods

### Time-restricted mouse matings and embryo processing

To perform time-restricted matings, a male and female mouse were placed together in a cage, beginning in the morning. Every hour thereafter, the female was visually checked for the presence of a vaginal plug. For embryo harvest, age was determined from the time of the observed plug, to the hour. For example, if a plug was first observed at 8:00AM, then the litter would be dissected 14 days later at 8:00AM for E14.0, at 11:00AM for E14.125, at 2:00PM for E14.25, etc. At the desired embryonic time point, pregnant female mice were euthanized using methods approved by the IACUC at the University of Kansas Medical Center. The embryos were harvested, washed in 1x PBS, decapitated, and fixed in 4% paraformaldehyde (PFA) overnight. The jaw was then removed, and the palate elevation status (bilaterally unelevated, unilaterally elevated, or bilaterally elevated) was recorded. Sex was determined by PCR, as described previously (Tunster, 2017).

### Static high-resolution magnetic resonance imaging of embryos *ex vivo*

Embryos from time-restricted matings were harvested at timepoints E14.0, E14.125, and E14.25 and fixed overnight in 4% PFA. The embryos were then incubated in 0.5 mM MnCl_2_ for at least 24 h, and placed in 1 × PBS in a 0.5 mL microcentrifuge tube for scanning. Scanning was performed at 9.4 T (Bruker Avance Neo system; Bruker, Billerica, MA, United States) using a 10 mm single loop surface RF coil to maximize sensitivity. High-resolution scans were acquired at a resolution between 20 × 25 × 170 µm and 40 × 50 × 170 µm.

### Segmentation and 3D reconstruction

Image segmentation of MRI scans was performed using ScanIP (Synopsis) 3D analysis software. Masks for the palatal shelves and tongue were created by manually tracing the structures in each frame of the MRI slice-package. The masks were then used to generate 3D renderings in the ScanIP software.

### Immunofluorescence

Embryo heads were fixed in 4% PFA overnight, processed for cryosectioning, and immunofluorescence was performed as described previously (Hall et al., 2020; Goering et al., 2021b). Tissue sections were incubated in primary antibodies KI-67 (CST, 12202) 1:500 and phospho-Myosin Light Chain Ser-19 (ECM Biosciences, MP4221) 1:100 overnight at 4°C, followed by secondary antibody Goat anti-Mouse Alexa-Fluor 594 (Invitrogen, A11037) 1:500 incubation for 2 h at room temperature, along with 0.1 μM DAPI. Images were obtained using confocal microscopy. Image quantitation and analyses were performed on the PS mesenchyme (excluding epithelium), which are described in detail in the supplement.

### Statistical analysis

To establish statistical significance, we calculated the quantitative measure for each independent sample (time-lapse recording, physical section of an embryo). The sets, containing 3–8 independent values, were compared by two-tailed Welch t-tests, which does not assume equal variance or paired data. For the calculations we used the scipy. stats.ttest_ind function of the python programming language.

## Results

### PS elevated between E14.0-E14.25 in C57BL/6J embryos

**FIGURE 1:**
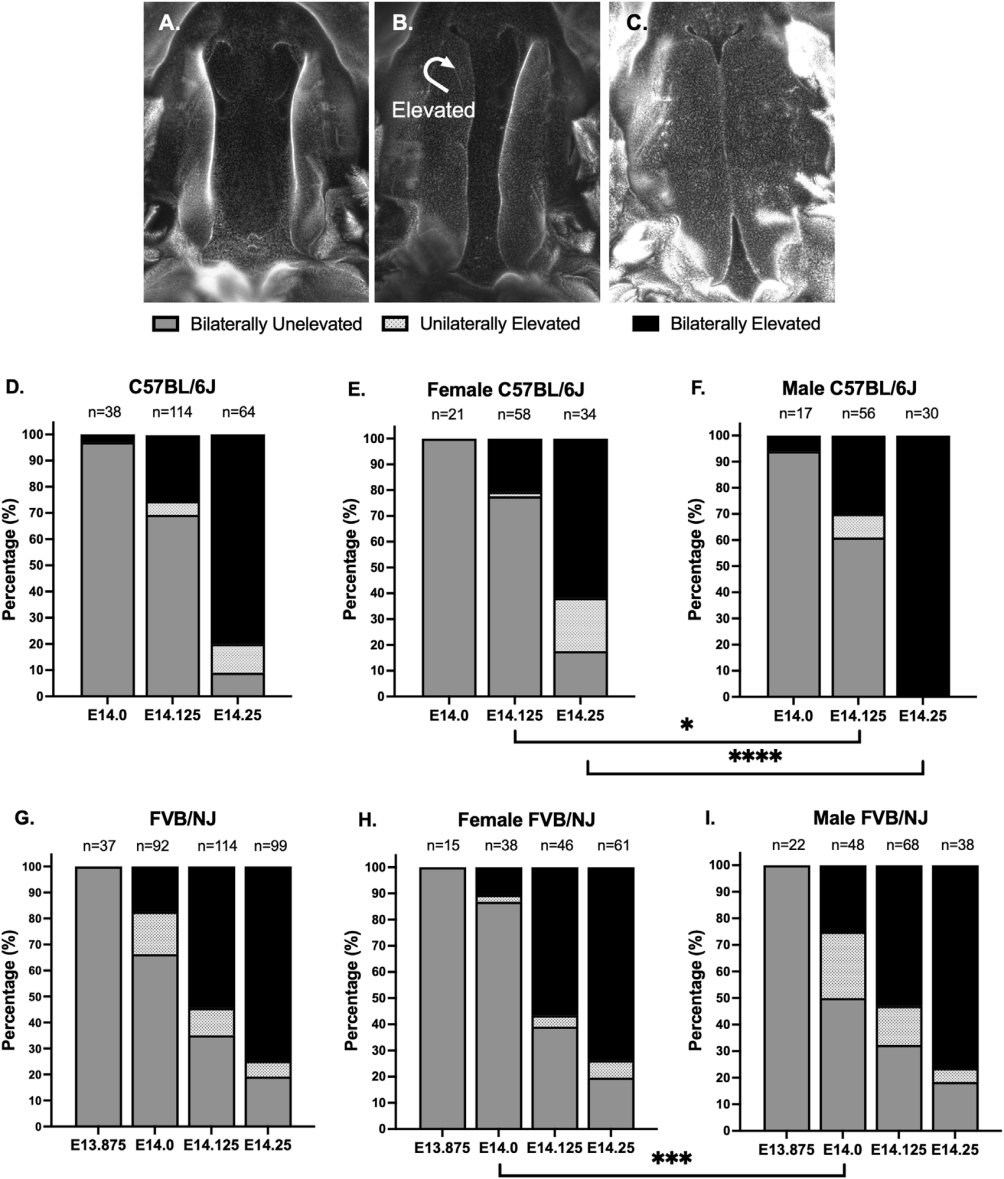
Time-restricted matings showed that palatal shelves elevated in less than 6 h. **(A–C)** To carefully track closure of palatal shelves (PS) in C57BL/6J and FVB/NJ embryos, we used time-restricted matings, checking for the presence of vaginal plugs every hour to reduce inter-litter variability in embryonic development. We scored the PS into 3 stages: bilaterally unelevated **(A)**, unilaterally elevated **(B)**, or bilaterally elevated **(C)**. Shown are DAPI-stained whole mount preparations, with the lower jaw and tongue removed. **(D)** We found that, at E14.0 in C57BL/6J embryos, only 3% (1/38) of PS were bilaterally elevated. At E14.25, just 6 h later, 80% (51/64) of embryos had bilaterally elevated PS, with an additional 11% (7/64) of embryos having unilaterally elevated PS. Finally, we found that, even at E14.125, 25% (29/114) of embryos had bilaterally elevated PS, indicating that, once initiated, the PS can complete elevation in less than 3 h. **(E, F)** We examined sex differences in the PS elevation of the isolated embryos. At E14.25, 100% (30/30) of male embryos **(F)**, but only 62% (21/34) of female embryos **(E)**, had bilaterally elevated PS (p < 0.0001, Fisher Exact Test). In fact, all C57BL/6J embryos that had bilaterally or unilaterally unelevated PS at E14.25 were female **(D)** vs. **(E)**. This sex difference was also observed at E14.125 (p < 0.04, Fisher Exact Test). **(G)** In comparison to C57BL/6J, the FVB/NJ embryos showed a broader window for PS elevation. At E14.0, 17% (16/92) of FVB/NJ embryos had bilaterally elevated PS and another 16% (15/92) had unilaterally elevated PS. At E13.875, 100% (37/37) of FVB/NJ embryos had bilaterally unelevated PS. At E14.25, 75% (74/99) of FVB/NJ embryos had bilaterally elevated PS, similar to the 80% observed in C57BL/6J embryos. **(H, I)** At E14.125 and E14.25, there were no sex differences observed in FVB/NJ embryos, however, there were significantly more bilaterally unelevated female embryos at E14.0 (p < 0.0005, Fisher Exact Test), suggesting an early milder delay that did not persist, in contrast to C57BL/6J embryos.

The most important aspect in studying PS elevation dynamics was to control the inter-litter variability in embryonic age. We focused on the timing of conception as a source of this variability. Standard timed matings call for housing the male and female mice together overnight (>16 h) and checking the vaginal plug in the morning, which can introduce significant inter-litter variability to embryonic age. Instead, we performed time-restricted matings by checking vaginal plugs every hour, which allowed us to determine embryonic age with a 1-h resolution. We began by identifying the latest embryonic timepoint at which the PS of C57BL/6J embryos were still vertical or unelevated. The convention in the field is to consider the E13.5-14.5 24-h period from overnight matings as the window for PS elevation, with PS at E13.5 as unelevated and PS at E14.5 as elevated. We wished to investigate the timing of PS elevation with a higher temporal resolution. We performed time-restricted matings, and scored embryos at various timepoints into three categories, or stages, of PS elevation: bilaterally unelevated (Figure 1A), unilaterally elevated (Figure 1B), or bilaterally elevated (Figure 1C). We found that, at E14.0, almost all (97%, 37/38) PS were bilaterally unelevated (Figure 1D, E14.0). At E14.25, 80% (51/64) of embryos had bilaterally elevated PS, while another 11% (7/64) had unilaterally elevated PS (Figure 1D, E14.25). Thus, our results indicated that, in C57BL/6J embryos, PS elevation is largely completed between E14.0 and E14.25, a period of less than 6 h.

To further assess the process of PS elevation, we looked at embryos at the intermediate E14.125 timepoint (Figure 1D, E14.125). 25% of embryos (29/114) had bilaterally elevated PS at this timepoint, implying that, once initiated, PS elevation needs less than 3 h to complete, as had been previously suggested by Walker and Fraser (Walker and Fraser, 1956). Interestingly, only 5% of embryos (6/114) had unilaterally elevated PS at E14.125, suggesting that it is not a stable intermediate state. Furthermore, unilateral elevation occurred with equal frequency to either the right or left PS, indicating a random process. Overall, our data depict PS elevation as a rapid embryonic process, which may or may not have an obligatory intermediate unilateral elevation state.

### PS elevation occurred later in female C57BL/6J embryos

We next wanted to determine if there were any sex differences in this PS elevation process in C57BL/6J embryos. At E14.25, 20% of embryos (13/64) had not yet completed PS elevation, including 9% (6/64) that had bilaterally unelevated PS and 11% (7/64) that had unilaterally elevated PS (Figure 1D, E14.25). Interestingly, at E14.25, all embryos with incomplete PS elevation were female, constituting 38% (13/34) of female embryos (Figure 1E, E14.25). In contrast, 100% of male embryos (30/30) at this timepoint had completed PS elevation (Figure 1F, E14.25), which was statistically significant (p < 0.0001, Fisher Exact Test). Earlier, at E14.125, 78% of female embryos (45/58) had not initiated PS elevation (Figure 1E, E14.125), compared to 61% of male embryos (34/56) (Figure 1F, E14.125), which was also statistically significant (p < 0.04, Fisher Exact Test). Thus, our data suggest that PS elevation occurred later in a significant proportion of female C57BL/6J embryos.

### PS elevation begins earlier and occurs over a longer period in FVB/NJ embryos

Strain differences in palate closure dynamics have been reported previously. These studies usually compared C57BL/6 strain to cleft palate susceptible strains such as A/J and S/Wyn, which invariably show delayed PS elevation (Walker and Fraser, 1956; Fraser, 1976; Diewert, 1982; Ciriani and Diewert, 1986; Diewert, 1986). We wanted to compare our observations in C57BL/6J to another common laboratory strain, FVB/NJ, that is not known to be susceptible to cleft palate. As before, we employed time-restricted matings and scored PS elevation at various timepoints. We found two main strain differences. First, PS elevation was initiated earlier in FVB/NJ compared to C57BL/6J embryos: at E14.0, 33% of FVB/NJ embryos (31/92) already had bilaterally or unilaterally elevated PS (Figure 1G, E14.0), compared to 3% (1/38) of C57BL/6J embryos (Figure 1D, E14.0). We confirmed that 3 h earlier, at E13.875, 100% of FVB/NJ embryos (37/37) had bilaterally unelevated PS (Figure 1G, E13.875). Second, in contrast to C57BL/6J embryos, we did not observe sex differences in PS elevation of FVB/NJ embryos at E14.125 or E14.25 (Figures 1H, I). Female FVB/NJ embryos did show a statistically significant delay in PS elevation compared to FVB/NJ males at E14.0 (p < 0.005, Fisher Exact Test), where 87% (33/38) of female embryos and 50% (24/48) of male embryos had bilaterally unelevated PS (Figures 1H, I). At E14.25, the overall extent of PS elevation was similar between C57BL/6J and FVB/NJ embryos, with 80% vs. 75% bilaterally elevated, respectively (Figures 1D, G).

### Anteroposterior dynamics of PS remodeling suggested a posterior to anterior elevation

**FIGURE 2:**
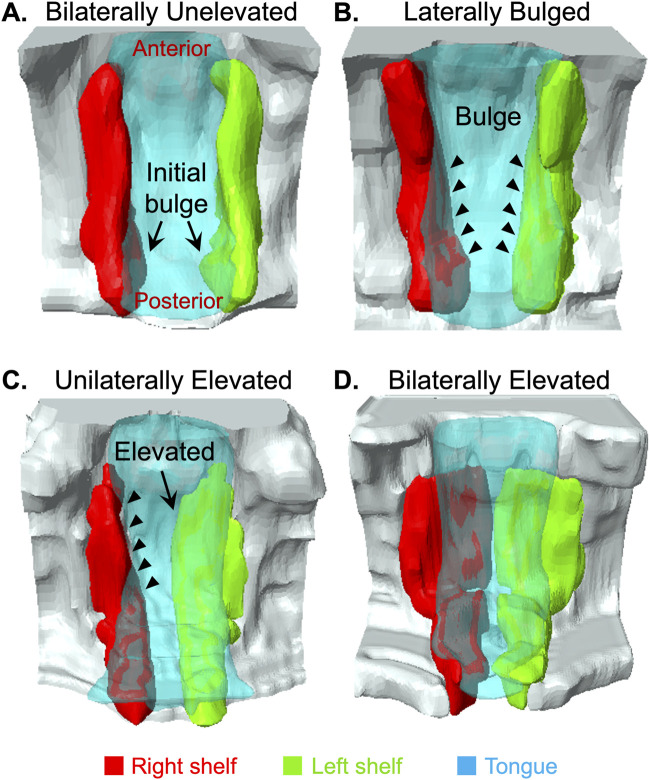
3D Imaging revealed anteroposterior dynamics of palatal shelf elevation. We used magnetic resonance imaging (MRI) scans of E14.125 embryos (n = 12) isolated at various stages of induction of palatal shelves (PS) elevation to segment and construct 3D images. Among the images of largely bilaterally unelevated PS, we noticed lateral bulges appearing bilaterally in the very posterior part of the PS [**(A)**, arrows]. In other scans, we observed more extensive lateral bulges [**(B)**, arrowheads]. Again, these bulges were most prominent in the posterior palate, and gradually tapered anteriorly [**(B)**, arrowheads]. We also scanned unilaterally elevated PS [**(C)**, arrow]. The unelevated PS in these instances showed the most prominent lateral bulge extending into the middle and anterior palate [**(B)** vs. **(C)**, arrowheads]. In this figure **(A–C)** clearly showed that the lateral bulges progressed from the posterior to anterior direction. Scans of bilaterally elevated PS **(D)** showed that adhesion first occurred in the anterior palate and proceeded posteriorly, as expected.

Previous studies have highlighted anteroposterior differences in PS elevation (Walker and Fraser, 1956; Yu and Ornitz, 2011; Bush and Jiang, 2012; Chiquet et al., 2016; Liu et al., 2021). Given our ability to consistently collect embryos in the process of PS elevation, we were able to scan and observe several C57BL/6J embryos (n = 12) using magnetic resonance imaging (MRI). The PS, tongue and portion of the maxilla in these scans were segmented, and 3D images were generated to better visualize the anteroposterior view during PS elevation. The 3D images showed that PS elevation does progress through lateral bulges above the tongue (Figure 2). However, these lateral bulges were first observed in, and were most prominent in, the posterior palate (Figure 2A, arrows), and the bulges gradually tapered anteriorly (Figure 2B, arrowheads). Figure 2B represents an example where the bulges were similarly extended bilaterally, with limited extension in the mid-palate region. Most instances of lateral bulges at the mid-palate region (Figure 2C, arrowheads) were in embryos with unilaterally elevated PS (Figure 2C, arrow). Interestingly, in embryos with unilateral elevation, the posterior-most ends of the PS all appeared to be already bilaterally horizontal (Figure 2C). Nonetheless, in all instances that we observed and consistent with current understanding (Bush and Jiang, 2012), PS adhesion began at a region anterior to mid-palate region, and extended posteriorly (Figure 2D). Taken together, these results showed that PS elevation–in contrast to adhesion–proceeded in the posterior to anterior direction.

### Regional changes in cell proliferation observed during PS elevation

**FIGURE 3:**
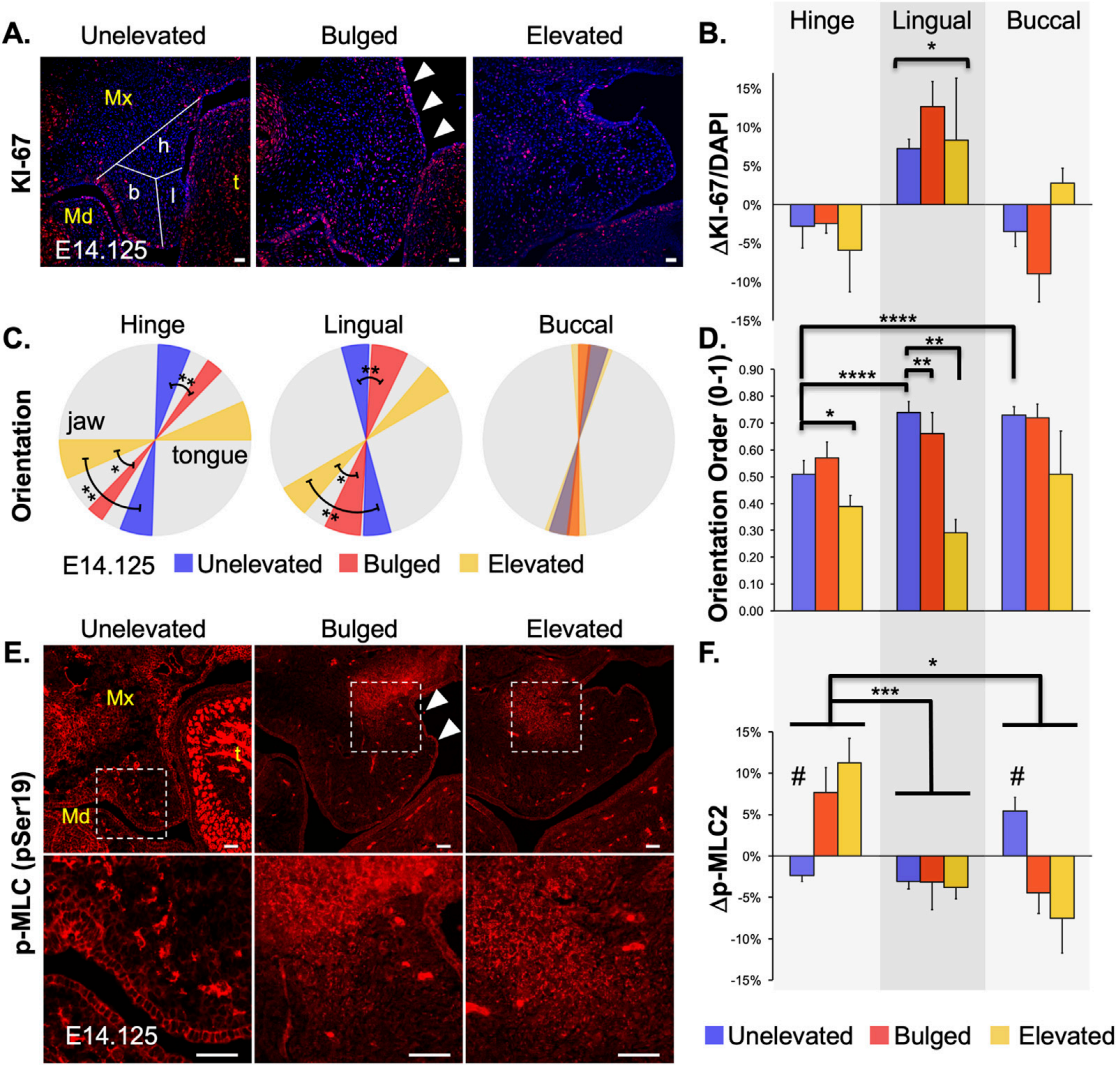
Changes in mesenchymal cell proliferation, orientation and actomyosin contraction during palatal shelf elevation. Cellular changes at mid-palate during elevation of palatal shelves (PS) were characterized by assessing mesenchymal cell proliferation **(A)**, cell orientation **(B)**, and phospho-Myosin Light Chain (p-MLC) level **(C)**. **(A)** Cell proliferation was assessed with anti-KI-67 antibody staining in cryosections from E14.125 embryos with unelevated (n = 8), bulged (arrowheads; n = 3), or elevated (n = 3) PS. **(B)** The entire PS area for each cryosection was analyzed to determine the mean number of cells positive for KI-67. Differences from the mean were then plotted for three regions of the PS: hinge (h), lingual (l), and buccal (b), as schematized in left panel in **(A)**. Only the lingual region, compared to hinge and buccal regions, showed a statistically significant (*p < 0.05) increase in cell proliferation in unelevated, bulged, and elevated PS. **(C)** Cell orientation was evaluated by analyzing the nuclear shape and angle. Regional averages and standard deviations were visualized using a wedge diagram. A horizontal tilt in the diagram indicated a predominant orientation along the lateral (tongue-jaw) axis, while a vertical tilt reflected alignment along the dorsal-ventral axis. The three regions of the PS (buccal, lingual, hinge) were analyzed separately (n = 4 for each). Compared with unelevated PS, the mesenchymal cells in the bulged and elevated PS become progressively more tilted towards the tongue in the hinge and lingual regions, suggesting a significant participation in the horizontal remodeling (*p < 0.03; **p < 0.01). Cells in the buccal region showed a very slight tilt towards the tongue, which did not change throughout PS elevation. Thus, cells in the buccal region do not appear to participate in the vertical-to-horizontal remodeling. **(D)** The extent of local cell orientation order, S, was also assessed for the samples in **(C)**. The orientational order decreased with the elevation process, and was lower in the hinge region (****p < 0.0001). **(E)** Activated p-MLC2 staining was assessed as a proxy for actomyosin activity in unelevated, bulged (arrowheads) and elevated PS. Qualitative changes showed increased expression in the buccal region of the unelevated PS, and in the hinge region of the bulged and elevated PS (boxed regions in top, magnified in lower panels). **(F)** Quantitative analysis was assessed as a change from average expression for each region (n = 4 for each). In the unelevated PS, spatially increased p-MLC2 expression was first observed in the buccal region (#p < 0.01). Later, in bulged and elevated PS, the increased expression was observed in the hinge region (*p < 0.02; ***p < 0.002). The lingual region showed consistently lower-than-average activity throughout PS elevation. Mx, maxilla; Md, mandible; t, tongue. Scale bars = 25 μm.

As mentioned earlier, an active role of cell proliferation in PS elevation has been debated. Once we could identify and capture the PS bulge region consistently, we looked at cell proliferation in the bulged PS using KI-67 immunostaining (Figure 3A). For analysis, we divided the PS coronal sections into 3 regions–lingual, buccal, and hinge–the last of which is the region in which the bulge develops (Figure 3A, left panel). Cell proliferation was significantly increased only in the lingual region, but this increase was present prior to elevation in the bilaterally unelevated PS and persisted through elevation (Figure 3B). This result suggested that increased cell proliferation within the bulge itself is not necessary for vertical-to-horizontal remodeling during PS elevation, but that increased cell proliferation in the lingual region may facilitate the process.

### Cell orientation changed drastically in the lateral bulge during PS elevation

We have previously shown that, at E13.5 (prior to PS elevation), the PS mesenchymal cells are aligned and slightly oriented towards the tongue (Goering et al., 2021a), potentially primed for PS elevation. Thus, we hypothesized cell orientation changes during PS elevation. We analyzed cell orientation by determining nuclear angle (Figure 3C) as well as extent of orientation order or homogeneity (Figure 3D). The nuclear angle in the hinge region of the unelevated PS was already tilted towards the tongue, compared to the lingual region (Figure 3C, blue; Supplementary Table 1), suggesting that the hinge region was primed for bulge formation. In the bulged PS, compared with unelevated PS, the orientation angles became more tilted in both the hinge and lingual regions (Figure 3C, red vs. blue). As expected, these angles became further tilted to almost horizontal in the elevated PS (Figure 3C, yellow vs. red). The cells in the buccal region did not show significant change in cell orientation, suggesting that these cells are largely excluded from the horizontal or elevated part of the PS (Figure 3C, Buccal; Supplementary Figure 1). Interestingly, in general, the extent of orientation homogeneity decreased in the regions where the cells were more tilted towards the tongue (Figure 3D). The cells in the buccal region, while not changing their angle during elevation, were highly ordered in their orientation. In contrast, the cells in the hinge region with the most tilt showed the lowest ordering. This suggests that the cell orientation within the PS subregion was either localized or patchy (Supplementary Figure 1).

### Sequential increase in activated myosin light chain levels in buccal and hinge regions during PS elevation

Next, we hypothesized that contractility via actomyosin activity was increased in the PS bulge region during PS elevation. Using immunofluorescence, we measured expression of phosphorylated myosin light chain (p-MLC), which participates in both muscle and non-muscle myosin-based contractility (Heisenberg and Bellaiche, 2013). We observed a two-step pattern in p-MLC levels (Figures 3E, F): in the unelevated PS, p-MLC was increased in the buccal region (Figure 3F; Buccal); later, in the bulged PS, p-MLC was instead increased in the hinge region, within the bulge (Figure 3F, Hinge); increased p-MLC in the hinge region persisted in the elevated PS. These PS regions with increased p-MLC staining also showed increased F-actin staining (Supplementary Figure 2), consistent with actomyosin contractility. A two-step pattern is consistent with an initial vertical PS contraction followed by a subsequent horizontal contraction, which together may propel the bulge in the horizontal direction.

## Discussion

Even though palatogenesis has been studied extensively, the actual timing and sequence of events during PS elevation remains unknown. In the field, the 24-h window between E13.5-E14.5 is generally assigned to PS elevation, even though studies have indicated that the process occurs much more rapidly than 24 h. Perhaps the most elegant and earliest of these studies were by Walker and Fraser (Walker and Fraser, 1956), who proposed that PS elevation *in utero* may take place in ∼3 h. Using more precise timed-matings with reduced inter-litter variability, we have now shown that, once initiated, PS elevation can be completed in as little as 3 h *in utero*. In C57BL/6J embryos, our data indicated that almost all PS elevation is completed within a developmental window of E14.0-E14.25 (6 h). This refined window should now allow investigators to more precisely identify changes in PS elevation dynamics in their respective transgenic cleft palate mouse models, which will help delineate the genetic networks at play in this process.

Strain differences have previously been studied in palatogenesis, particularly for strains that showed increased occurrence of cleft palate. In general, these studies showed a delay in palate elevation in strains susceptible to cleft palate, e.g., A/J and A/WySnJ, compared to C57BL/6 (Walker and Fraser, 1956; Fraser, 1976; Diewert, 1982; Ciriani and Diewert, 1986; Diewert, 1986). We compared C57BL/6J to the FVB/NJ strain, which is not reported to have increased susceptibility to cleft palate. We found that the overall window of PS elevation is broader and begins earlier in FVB/NJ embryos (∼9 h; E13.875-E14.25) compared to C57BL/6J embryos (∼6 h; E14.0-E14.25). However, at E14.25, a similar number of embryos had completed PS elevation in both strains.

A surprising finding was the observation of sex differences in the timing of PS elevation. Isolated cleft palate in humans is known to occur more frequently in females than in males (∼2:1) (Mossey et al., 2003; Mossey et al., 2009), and some early studies suggesting delayed PS elevation in female human (Burdi and Silvey, 1969) and mouse (Burdi and Faist, 1967) embryos. We found that only 61% (21/34) of C57BL/6J female embryos had completed PS elevation by E14.25, compared to 100% of C57BL/6J male embryos (30/30). This result raises the possibility that the C57BL/6J background may predispose females to cleft palate. Consistent with this hypothesis, we previously reported that in a mouse model of SPECC1L gain-of-function (*Specc1l* ^*DCCD2*^), on a mostly C57BL/6J background (N3 generation) mixed with some FVB/NJ, the cleft palate phenotype among heterozygotes was more prevalent in female (17%) than male (5%) embryos (Goering et al., 2021b). However, we emphasize that we are not proposing that the FVB/NJ background offers any protection against cleft palate. In fact, our unpublished data suggest a similar overall incidence of cleft palate in *Specc1l* ^*DCCD2/+*^ heterozygotes (∼15%) on a pure FVB/NJ background (>N8 generation), albeit with equal occurrence in male or female embryos (not shown). Thus, sex differences in PS elevation represent one of many factors that affect palatogenesis and need to be considered carefully in future studies.

Our data showed that the PS undergo vertical-to-horizontal remodeling via lateral bulge formation. Moreover, our imaging of intermediate states indicated that the bulges originated in the posterior palate, and gradually extended anteriorly, consistent with Walker and Fraser (1956). In contrast, PS adhesion eventually began in the anterior to mid-palate region and extended posteriorly, as expected.

Walker and Fraser (1956) further suggested that the unilateral PS elevation is an “obligate” intermediate step. This would imply that one PS elevates above the tongue before the second, and that, to accommodate this movement, the tongue must go through a rocker-like motion. Some subsequent studies observed unilaterally elevated PS in normal embryos (Yu and Ornitz, 2011; Bush and Jiang, 2012; Liu et al., 2021), and others that observed unilateral elevation in mutant mouse models of cleft palate, including those with *Specc1l* deficiency (Liu et al., 2008; Hill et al., 2015; Butali et al., 2019; Goodwin et al., 2020; Hall et al., 2020). We not only observed many instances of unilateral PS elevation, but also observed several embryos with equally bilateral bulges from unelevated PS. When we compared these two occurrences, the unelevated shelf in embryos with unilateral PS elevation had a more anteriorly progressed bulge. Thus, we argue that the bulges initially developed bilaterally, and when they progressed to a certain extent, unilateral elevation took place. We did not find any preference for right or left PS elevation among unilaterally elevated samples. Thus, if unilateral elevation is indeed an “obligate” intermediate step, it is very transient, and happens late in the elevation process. A normal palate closure process that proceeded unilaterally (one PS at-a-time) would be more consistent with observations in humans, as occurrences of unilateral human clefts are common (Mossey et al., 2009). Further, a mechanical force generated from the posterior regions of the palate may not be evenly split, and a one-at-a-time PS elevation may allow for more flexibility, and reduce the force required to displace the tongue.

**FIGURE 4:**
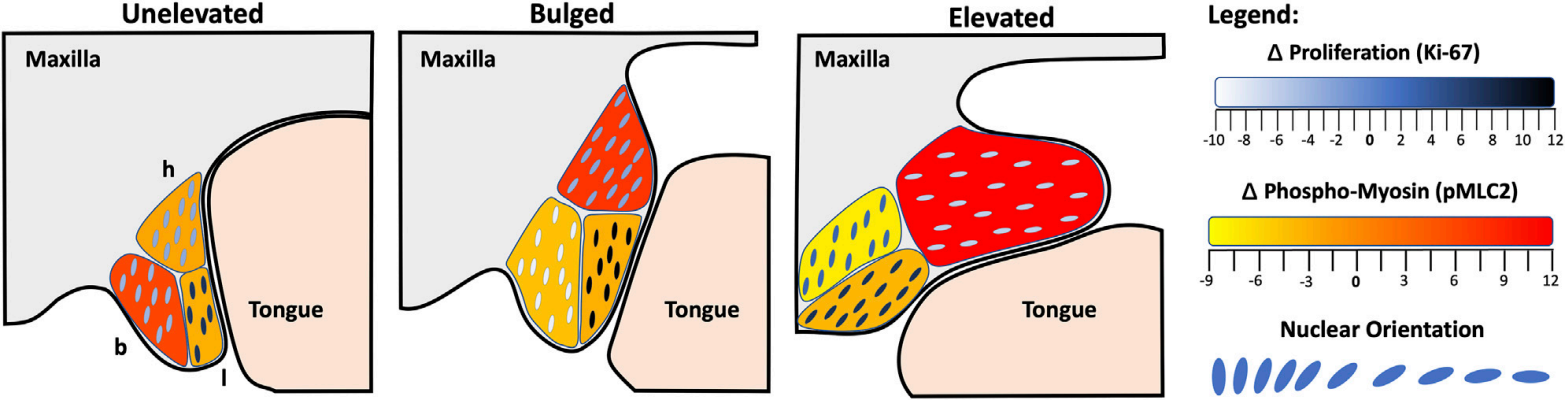
Model of palatal shelf elevation. Schematic summarizing the cellular changes observed in the buccal (b), lingual (l) and hinge (h) regions of the unelevated, bulged, and elevated palatal shelves (PS). In the unelevated PS (left), we observed increased cell proliferation in the lingual region, a slight tilt in orientation towards the tongue in both the buccal and hinge regions, and increased actomyosin activity in the buccal region. The latter may participate in vertical contraction of the PS. In the bulged PS (middle), increased cell proliferation persisted in the lingual region, cell orientation was acutely tilted towards the tongue within the bulge in the hinge region, and actomyosin activity was now increased in the hinge region. The acute orientation and increased actomyosin activity in the hinge region may participate in the propulsion of the bulge in the horizontal direction. In the elevated PS (right), cell proliferation continued in the posterior lingual region, the cell orientation in the lingual and hinge regions were almost horizontal, and actomyosin activity was still increased in the hinge region. The lack of change in the cell orientation in the buccal region suggests that cells in that region did not participate in the vertical-to-horizontal remodeling. The spatial pattern of actomyosin activity suggests that vertical PS contraction proceeded in the buccal region, followed by dorsal contraction in the hinge region, coinciding with the horizontal bulge formation. It remains to be seen whether the ventral portion of the hinge region and proliferating lingual cells are the ones that are propelled into the elevated PS.

Lastly, the nature of PS elevation has long been debated (Lazzaro, 1940; Walker and Fraser, 1956; Ferguson, 1977; 1988; Bush and Jiang, 2012). The vertical-to-horizontal remodeling was thought to involve rotation as well as cell proliferation. While Walker and Fraser (1956) disagreed with both, it remained to be seen if cell proliferation played a role. We now show that, during elevation, PS bulges in the hinge region do not show any relative increase in cell proliferation (Figure 4). However, we did observe increased cell proliferation in the lingual region, which was already present in the unelevated PS, and persisted in the elevated PS. Since the lingual region is immediately ventral to the hinge region where the bulges form, the increased cell proliferation may passively support PS elevation. This also suggests that there is no horizontal growth following elevation *per se*; instead, the vertical growth prior to PS elevation persists in the lingual region and culminates with PS elevation and adhesion.

Our data are consistent with the findings of Chiquet et al. (2016), who previously showed that in the mesenchyme of medial PS, immediately prior to elevation (bulged), there are elongated nuclei oriented towards the tongue. They also showed strong expression of F-actin in these PS, suggesting a role for actin-based contraction in PS elevation. We have now provided quantitative evidence for both an acute mesenchymal cell orientation towards the tongue and an increase in activated p-MLC staining, parsed into three subregions of the medial PS (Figure 4). We also observed increased F-actin staining in the same PS regions that showed increased p-MLC staining (Supplementary Figure 2). Thus, bolstering the argument that cell orientation and actomyosin forces participate in the rapid movement of PS bulges during elevation. Following PS elevation, the lingual and hinge regions showed almost horizontal cell orientation, as expected, but the buccal region cells did not. This latter result suggested that the buccal region did not participate in the horizontal remodeling. Instead, we observed an increase in activated myosin light chain staining in the buccal region of the unelevated PS, suggesting that the buccal region may participate in the vertical contraction of the PS as the lateral bulges appeared. The combination of cellular orientation towards the tongue, coordinated actomyosin contractility, and permissive extracellular matrix conditions, together allow for rapid reorientation of the shelves above the tongue. To help further delineate this process, future studies should investigate mouse mutants with defects in PS elevation, cell orientation or alignment, and actomyosin contractility. We assert that PS elevation is the most dynamic and sensitive step in palatogenesis, and is especially susceptible to both genetic and environmental factors in the etiology of cleft palate.

## Data Availability

The original contributions presented in the study are included in the article/Supplementary Material, further inquiries can be directed to the corresponding author.
