# Supplementary material for: Novel insights into palatal shelf elevation dynamics in normal mouse embryos
Source: Front Cell Dev Biol. 2025 Feb 11;13:1532448. doi: 10.3389/fcell.2025.1532448 (PMC11850390; doi:10.3389/fcell.2025.1532448)
Supplement: Supplementary file 1 [file DataSheet1.docx]

Supplementary Material

**1 Supplementary methods**

**1.1 Sex determination PCR**

Yolk sacs were taken at the time of harvesting for genotyping. Sex was determined by PCR, using primers flanking an 84 bp deletion of the *Rbm31x* gene relative to its gametolog *Rbm31y* (Forward: CACCTTAAGAACAAGCCAATACA; Reverse: GGCTTGTCCTGAAAACATTTGG). A single product (269 bp) was amplified in female embryos and two products (269 bp and 353 bp) were amplified in male embryos (Tunster, 2017).

**1.2 Whole-mount DAPI staining**

Whole-mount DAPI staining of the palate was achieved by decapitating fixed embryos, removing the lower jaw, and incubating the exposed palates in 500-1000 nM DAPI solution overnight. The palates were then imaging on a Nikon SMZ 1500 stereomicroscope (Sandell et al.; Goering et al., 2021a; Goering et al., 2021b).

**1.3 Image quantitation and analyses**

Left or right aspects of DAPI-stained palatal sections of embryos at E14.0, E14.125, and E14.25 were categorized as unelevated, bulged, or elevated. Confocal micrographs taken with a 20x objective were analyzed by the following procedure: first, the palatal mesenchyme was delineated manually using the ImageJ "Polygon selection" tool; subsequent analyses were restricted to the selected region of interests. Second, the hinge (h), lingual (l), and buccal (b) aspects of each palate sample were delineated manually using the ImageJ "multi-point" tool, by placing a control point to the center of each of the three regions. For the boundaries of the hinge, lingual, and buccal regions, we considered the Voronoi partition of the image, generated by the three manually selected control points. Thus, as an example, we included an image pixel into the "hinge" region, if its distance to the "hinge" control point was less than its distance to the other two.

As no well-established classification of the palatal shelf (PS) sub-regions exists in the literature, in the manual marking procedure we relied on the following histological landmarks. (i) The buccal/lingual boundary divides the distal aspect of the PS into two domains of roughly equal size. In an unelevated PS this boundary passes through the tip of the shelf, while in an elevated PS the buccal domain is the dorso-lateral aspect of the elevated PS. (ii) We assumed that the boundary between the hinge and lingual domains bisects the lingual PS epithelium into two halves of equal length. (iii) Finally, the boundary between the buccal and hinge regions is already determined by the three control points needed to establish the other two boundaries. We expect that the imprecise delineation of these regions contributed to the variability of the derived quantities, thus reducing the likelihood of finding statistically significant differences. Following are representative images of E14.125 unelevated, bulged and elevated PS showing the assigned boundaries.


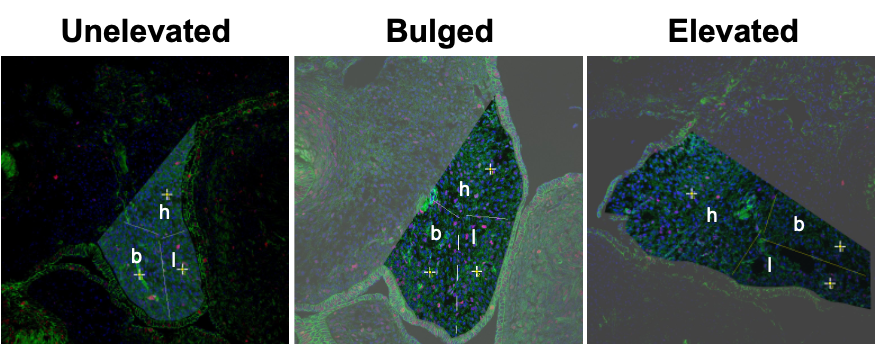
Spatial differences in marker fluorescence were studied by calculating the average fluorescence intensity (*F*) for each of the hinge, buccal, and lingual regions, as well as for the entire palate (*F_0,_* the sum of the areas encompassing each of the three regions). The relative excess or sanctity of the marker was then calculated by the ratio $\frac{F}{F_{0}}$. Accordingly, for each region, spatial differences in phospho-Myosin Light Chain were characterized by calculating:

$$\Delta p\text{-}MLC2=\frac{F(p\text{-}MLC2)}{F_{0}(p\text{-}MLC2)}$$

for each region. Cell proliferation is characterized by the fraction of KI-67-positive nuclei; to characterize differences in proliferation, we normalized KI-67 differences by the local abundance of DAPI-stained nuclei using:

$$\Delta KI\text{-}67=\frac{{F(KI\text{-}67)}/{F_{0}(KI\text{-}67)}}{{F(DAPI)}/{F_{0}(DAPI)}}$$

**1.4 Nuclear orientation assay**

To quantitatively characterize local orientational ordering of mesenchyme nuclei, we performed the following image analysis sequence: 1) DAPI-labeled frozen sections were imaged with 20x objective magnification; 2) using ImageJ (Schindelin et al.), the palatal shelf area was delineated, then nuclei were segmented by brightness-based global thresholding; 3) touching nuclei in the binary segmented image were resolved by a watershed transformation; 4) segmented clusters larger than 10 pixels were identified and fitted with an ellipse; 5) nuclei with an unambiguous orientation (the ratio of the minor and major ellipse axes being less then 0.8) were assigned into cells of a spatial grid with a cell size of 50 μm; 6) in each cell, the scalar 2D nematic order parameter (Das et al.) was calculated as $S^{2}= \left\langle cos2\theta\right\rangle^{2} + \left\langle sin2\theta\right\rangle^{2}$, where theta ($\theta$) is the angle between the long axis of the ellipse and a reference direction, and the averages $\left\langle.. \right\rangle$ were calculated for each nucleus within the grid cell. The order parameter S=1 indicates a configuration in which nuclei are oriented completely parallel, while S=0 indicates that the nuclei are oriented completely randomly. Values 0<S<1 indicate various degrees of partial ordering. Using the same data, we also calculated the direction of the local prevailing order. We then used the same measures to compare regions within the palatal shelves by pooling the segmented nuclei from multiple specimens into three spatial domains (lingual, buccal, and hinge).

**1.5 Supplementary Methods References**

Das, R., Kumar, M., and Mishra, S. (2017). Order-disorder transition in active nematic: A lattice model study. *Sci Rep* 7(1)**,** 7080. doi: 10.1038/s41598-017-07301-w.

Goering, J.P., Isai, D.G., Hall, E.G., Wilson, N.R., Kosa, E., Wenger, L.W., et al. (2021a). SPECC1L-deficient primary mouse embryonic palatal mesenchyme cells show speed and directionality defects. *Scientific Reports* 11(1)**,** 1452. doi: 10.1038/s41598-021-81123-9.

Goering, J.P., Wenger, L.W., Stetsiv, M., Moedritzer, M., Hall, E.G., Isai, D.G., et al. (2021b). In-frame deletion of SPECC1L microtubule association domain results in gain-of-function phenotypes affecting embryonic tissue movement and fusion events. *Hum Mol Genet* 31(1)**,** 18-31. doi: 10.1093/hmg/ddab211.

Sandell, L., Inman, K., and Trainor, P. (2018). DAPI Staining of Whole-Mount Mouse Embryos or Fetal Organs. *Cold Spring Harb Protoc* 2018(10). doi: 10.1101/pdb.prot094029.

Schindelin, J., Arganda-Carreras, I., Frise, E., Kaynig, V., Longair, M., Pietzsch, T., et al. (2012). Fiji: an open-source platform for biological-image analysis. *Nat Methods* 9(7)**,** 676-682. doi: 10.1038/nmeth.2019.

Tunster, S.J. (2017). Genetic sex determination of mice by simplex PCR. *Biol Sex Differ* 8(1)**,** 31. doi: 10.1186/s13293-017-0154-6.

# 2 Supplementary Figures and Table

#
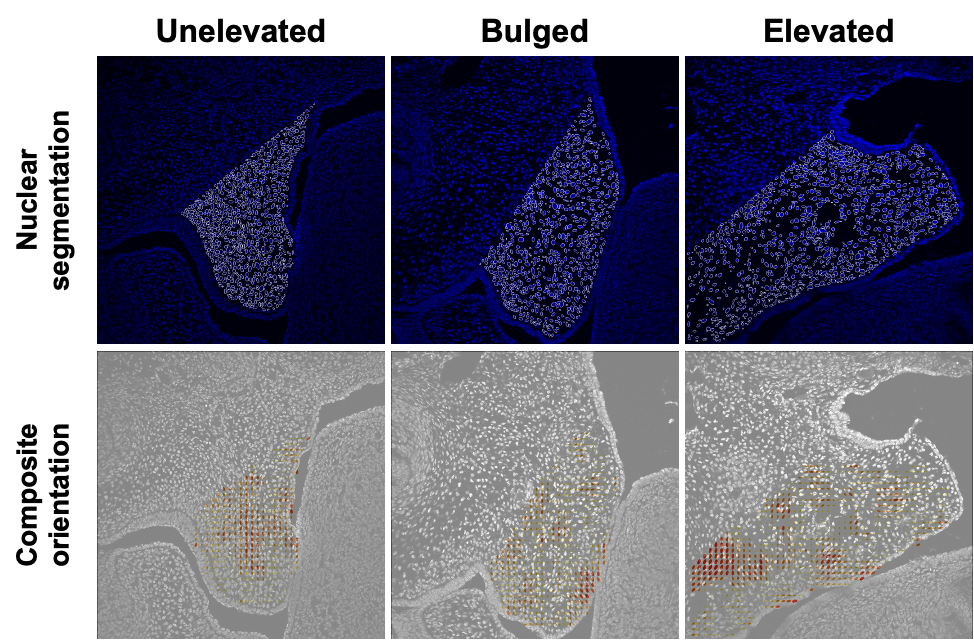
2.1 Supplementary Figures

# Supplementary Figure 1. Nuclear orientation analysis. Top row: DAPI-stained nuclei were segmented through brightness thresholding within the palatal shelf (PS) region of the confocal slice. Each segmented cluster was fitted with an ellipse, outlined in white. Bottom row: The local average orientation of the segmented nuclei, S, was visualized as a heatmap superimposed on the corresponding grayscale confocal image.  Colors range from deep red (S=1, indicating complete alignment) and light yellow (S=0, indicating random orientation). The heatmap is presented using ellipses arranged on a grid, where the ellipse orientation reflects the direction of the local alignment. Representative samples are shown for an unelevated (left), bulged (center) and elevated (right) PS.

**
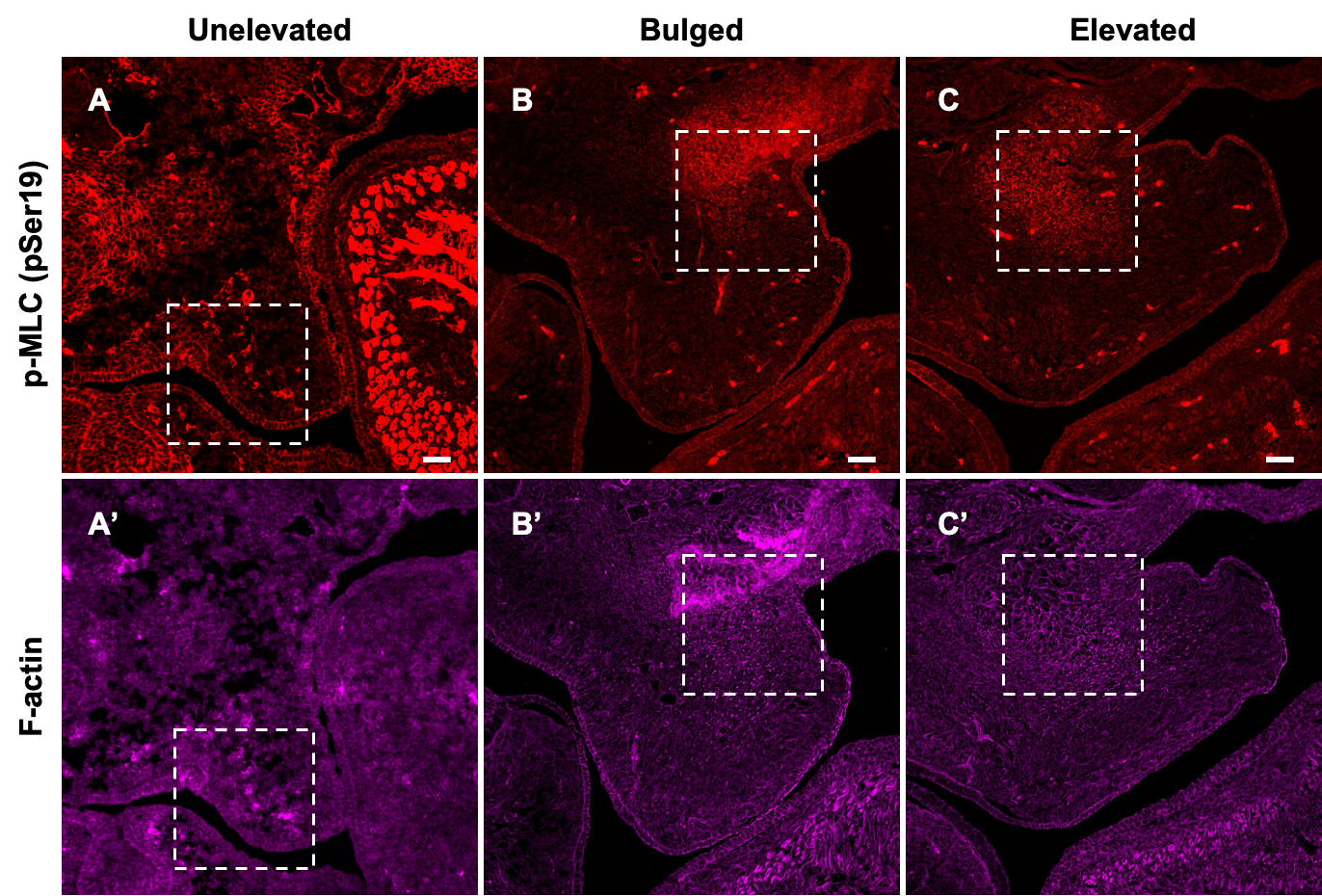
**

**Supplementary Figure 2.** Phosphorylated myosin light chain (p-MLC; A-C) co-staining with filamentous actin (F-actin; A’-C’) in E14.125 sections through unelevated (A, A’), bulged (B, B’) and elevated (C, C’) palatal shelves shown in Figure 3D. The regions with increased p-MLC staining (boxed as in Fig.3D) also show increased F-actin staining.

**2.2 Supplementary Table 1. Statistical comparisons for mesenchymal cell orientation angles shown in Figure 3C.**
